# Remifentanil and Neonatal Intubation: Balancing Efficacy and Safety

**DOI:** 10.3390/children13091169

**Published:** 2026-08-29

**Authors:** Lara Garabedian, Gerbrich E. van den Bosch, Sophie Vanhaesebrouck, Karel Allegaert

**Affiliations:** 1 Department of Neonatal Intensive Care, Ghent University Hospital, 9000 Ghent, Belgium; lara.garabedian@uzgent.be (L.G.); sophie.vanhaesebrouck@uzgent.be (S.V.); 2 Department of Neonatal and Pediatric Intensive Care, Division of Neonatology, Erasmus MC—Sophia Children’s Hospital, 3015 GD Rotterdam, The Netherlands; g.vandenbosch@erasmusmc.nl; 3 Clinical Pharmacology and Pharmacotherapy, Department of Pharmaceutical and Pharmacological Sciences, KU Leuven, 3000 Leuven, Belgium; 4 Department of Development and Regeneration, KU Leuven, 3000 Leuven, Belgium; 5 Department of Hospital Pharmacy, Erasmus University Medical Center, 3015 GD Rotterdam, The Netherlands

**Keywords:** remifentanil, neonatal intubation, safety, pharmacokinetics, opioids

## Abstract

**Highlights:**

**What are the main findings?**

- In this narrative review, we synthesize the clinical evidence on remifentanil for neonatal intubation, with emphasis on efficacy, safety, dosing, administration technique, and its potential role in INSURE or LISA procedures.
- Our current practices are informed by a combination of small clinical trials, observational studies, and pharmacological research.

**What are the implementations of the main findings?**

- Remifentanil may be a useful option in selected neonatal intubation settings, particularly when rapid recovery is desired, but the available evidence is limited and heterogeneous. However, vigilance regarding chest wall rigidity and close monitoring are required. Further research is warranted to compare remifentanil with other opioids that have a similar pharmacological profile.

**Abstract:**

Background: Endotracheal intubation is a painful and stressful procedure for neonates, and premedication is recommended for non-emergent settings. However, there is no consensus on the optimal pharmacological regimen. In recent years, several drugs have emerged as treatment options to improve comfort and safety of neonates undergoing endotracheal intubation. Worldwide, a range of agents are used. Remifentanil is a short-acting opioid with rapid onset and recovery characteristics that may be useful for neonatal intubation. Methods: A structured search of PubMed/MEDLINE, Embase, and Cochrane Library databases (1990–December 2024) identified publications evaluating remifentanil for neonatal intubation. Clinical studies, pharmacokinetic investigations, reviews and guidelines were qualitatively synthesized with an emphasis on dosing, efficacy, safety, dosing, administration technique, and clinical context. Results: The literature search retrieved 45 publications, but only a small subset were randomized clinical studies. Findings generally support feasibility and short-term efficacy, while results vary across studies, particularly for intubation conditions and respiratory adverse events. Chest wall rigidity, apnea, and desaturation appear to be influenced by dose, administration rate, patient characteristics, and co-medication, although the available studies are too small and heterogeneous to define an optimal regimen. Conclusions: Remifentanil can be considered for selected non-emergent neonatal intubation and INSURE procedures when administered by experienced teams under appropriate monitoring conditions. Particular caution is warranted in clinically unstable infants, during LISA procedures where preservation of spontaneous breathing is desirable, and in settings where immediate airway rescue capabilities are unavailable. The study did not demonstrate clear superiority of remifentanil. Current evidence is limited and heterogeneous, and larger comparative studies with standardized dosing, administration, and outcome definitions are needed.

## 1. Introduction

Endotracheal intubation is painful and stressful for neonates and may cause bradycardia, hypoxia, and other physiological disturbances [1,2,3,4,5]. Premedication is recommended for non-emergent neonatal intubation because it can reduce procedural stress and improve intubation conditions [5,6,7]. More importantly, Premedication has been shown to reduce pain and discomfort experienced by the neonate during the procedure and improve intubation conditions [5,6,7,8,9]. Early-life exposure to pain and stress is associated with adverse long-term neurodevelopmental and physiological outcomes in neonates [10].

In 2010, the American Academy of Pediatrics (AAP) recommended routine premedication for all non-emergent neonatal intubations, alongside careful preparation and supportive non-pharmacological measures to minimize instability and procedural trauma [5,8,9,11].

However, there is no consensus on the optimal pharmacological regimen [5]. Current guidance generally supports a combination of analgesia, sedation when required, and—depending on the clinical context—neuromuscular blockade and vagolytic therapy [8,9]. The French recommendations favor propofol or an opioid plus neuromuscular blocker for tracheal intubation, whereas propofol is preferred for Less Invasive Surfactant Administration (LISA); the 2026 Canadian guidance recommends a vagolytic, rapid-acting opioid, and short-acting neuromuscular blocker for endotracheal intubation and INSURE [12,13].

The choice of agent should reflect the procedure, the infant’s clinical condition, the need for spontaneous breathing, and the expected duration of airway manipulation. Opioids therefore remain central due to their analgesic efficacy [14]. Morphine is familiar but has a slow onset and prolonged duration, limiting its usefulness in urgent or short procedures [14,15]. Fentanyl offers faster onset but carries risks of chest wall rigidity and respiratory depression, particularly with rapid administration [1]. Midazolam improves sedation but lacks analgesia and has been associated with hypotension and potential neurodevelopmental concerns, limiting its role as a sole agent [14]. Propofol produces excellent intubation conditions with rapid recovery but may cause significant hypotension, particularly in very preterm or unstable neonates and has no analgesic effect [16,17].

Neuromuscular blocking agents improve intubation success and reduce airway trauma when combined with adequate analgesia and sedation [18]. Succinylcholine has rapid onset but potential serious adverse effects, whereas rocuronium offers a safer profile at the cost of longer paralysis [19,20]. Muscle relaxants should never be used alone. Vagolytics, such as atropine, are frequently used to prevent procedure-related bradycardia, particularly in preterm infants, although routine use remains debated [21].

The growing interest in remifentanil stems from its unique pharmacokinetic profile, characterized by a very rapid onset (approximately 1 min) and an extremely short half-life due to metabolism by non-specific plasma and tissue esterases [22,23,24,25]. In contrast, other potent synthetic opioids such as fentanyl and sufentanil are highly lipophilic compounds with longer context-sensitive half-times due to redistribution and accumulation in peripheral tissues, while alfentanil, although shorter-acting than fentanyl, still exhibits slower clearance than remifentanil [26,27]. However, remifentanil is not without risks. Reported side effects include respiratory depression, apnea, bradycardia, and chest wall rigidity, similar to other opioids [23,28,29]. Because of its potency and rapid action, careful dosing and close monitoring are essential. Additionally, its very short duration may require coordination with other agents (e.g., sedatives or muscle relaxants) to maintain adequate conditions throughout the procedure.

This review focuses on the clinical evidence for efficacy and safety, dosing and administration, and the circumstances in which remifentanil may or may not be a reasonable option for neonatal intubation.

## 2. Materials and Methods

### 2.1. Literature Search

This narrative review was conducted to evaluate the use of remifentanil for neonatal intubation, focusing on its pharmacological properties and clinical applicability. A comprehensive literature search was performed using PubMed/MEDLINE, Embase, and the Cochrane Library from 1990 through December 2024. Search terms included “remifentanil” or “ultra-short-acting opioid” in combination with “neonate”, “newborn”, or “preterm infant”, and “intubation”, “endotracheal intubation”, or “INSURE”, together with terms such as “premedication”, “sedation”, “analgesia”, “muscle relaxant”, and “pharmacokinetics and dynamics”.

Eligibility criteria were defined before study selection. Studies were included if they involved neonates or preterm infants undergoing endotracheal intubation or INSURE procedures, evaluated remifentanil as a sedative, analgesic, or premedication agent, and reported efficacy, safety, pharmacokinetic, pharmacodynamic, or other clinically relevant outcomes related to neonatal airway management.

Eligible study designs included randomized controlled trials, observational studies, cohort studies, pilot studies, systematic reviews, clinical practice guidelines, consensus statements, and pharmacological reviews. Studies were excluded if they did not include neonatal populations, evaluated remifentanil exclusively in older children or adults without direct neonatal relevance, were conference abstracts lacking sufficient methodological detail, or represented duplicate publications of the same dataset.

Given that the manuscript is a narrative review, titles, abstracts and full texts were screened by a single reviewer, and data were extracted by the same reviewer. Data extraction included study design, patient population, remifentanil dosing regimen, comparator interventions, intubation conditions, adverse events, and reported conclusions regarding efficacy and safety. Only articles published in English were considered for inclusion. The reference lists of all included articles were screened to identify additional relevant studies.

### 2.2. Quality of Evidence and Risk of Bias

The evidence was interpreted according to study design and methodological limitations rather than pooled quantitatively. Randomized trials were considered more informative for comparative efficacy, whereas observational and pilot studies were used mainly to assess feasibility, dosing, and safety signals. Across the literature, small sample sizes, single-center designs, subjective outcome measures, and differences in gestational age, clinical status, operator experience, co-medications, and administration technique limit certainty and direct comparability. The highest level of evidence was provided by randomized controlled trials conducted by Pereira e Silva et al. [15], Choong et al. [30], Badiee et al. [11], and Avino et al. [14], which directly evaluated remifentanil as premedication for neonatal endotracheal intubation. These studies represent the strongest available evidence regarding efficacy and short-term safety in neonatal populations. Additional evidence was derived from prospective pilot studies and observational cohorts, including investigations by Welzing et al. [31], Smith et al. [32], de Kort et al. [33] and Audil et al. [34]. While these studies contributed valuable information regarding feasibility and safety, their interpretability was limited by relatively small sample sizes and non-randomized study designs.

Several potential sources of bias were identified. Selection bias was common due to the predominantly single-center nature and limited sample sizes of neonatal studies, which may restrict generalizability. Performance bias was possible because blinding of clinicians performing intubation was frequently impractical given the distinct clinical effects and administration methods of the study medications. Detection bias may also have been present, as outcomes such as intubation conditions, sedation quality, and ease of intubation often relied on subjective assessment.

Observational studies were particularly vulnerable to confounding from differences in gestational age, severity of illness, operator experience, concomitant atropine administration, and the use or omission of neuromuscular blocking agents. Finally, publication bias cannot be excluded, as the relatively small body of literature raises the possibility that negative or inconclusive studies remained unpublished. Collectively, these limitations should be considered when interpreting the available evidence and highlight the need for larger, multicenter randomized trials using standardized protocols and outcome measures.

Risk-of-bias assessment was performed to facilitate interpretation of the available evidence and the limited number of eligible publications resulted in inclusion of the available studies evaluating remifentanil for neonatal intubation.

Risk of bias was assessed according to study design. It was performed by one reviewer. Randomized controlled trials were evaluated using the Cochrane Risk of Bias 2 (RoB 2) tool [35], while non-randomized studies were assessed using the Newcastle–Ottawa Scale (NOS) [36]. Each criterion was coded green (criterion met), orange (criterion partially met or unclear) or red (criterion not met). Only green ratings contributed to the quality score. Studies meeting at least seven criteria were considered of sufficient methodological quality. Given the limited number of available studies, all publications evaluating the use of remifentanil for endotracheal intubation were included in the final analysis.

## 3. Results

### 3.1. Number and Types of Studies

After applying the predefined eligibility criteria, the literature search yielded 45 publications for inclusion in the final evidence base, comprising randomized controlled trials, observational studies, pharmacokinetic investigations, pilot studies, systematic reviews, consensus statements, and clinical practice guidelines. Owing to the narrative nature of this review, no formal meta-analysis or statistical pooling of data was performed. The key findings are summarized in Table 1.

### 3.2. Physiochemical Characteristics and Unique Pharmacokinetics of Remifentanil (See Figure 1)

Remifentanil, 3-(4-methoxycarbonyl-4-[(L-oxopropyl)-phenylamino]-L-piperidine) propanoic acid methyl ester, is a synthetic opioid similar to other piperidine derivatives [23]. Its mechanism of action involves providing potent analgesia alongside a mild sedative effect [22]. Its pharmacodynamic profile is consistent with that of other μ-opioid receptor agonists [37]. Receptor binding studies have shown that remifentanil has a strong affinity for μ (mu) opioid receptors and lesser affinity for other opioid receptors, such as κ (kappa) and σ (sigma) [23,38]. It shows minimal interaction with non-opioid receptors and is known to be competitively antagonized by naloxone [28,39].

However, the distinctive properties of remifentanil relate to its unique pharmacokinetic profile [22,28]. These characteristics make it particularly suitable for clinical situations requiring rapid onset and offset of action. The most notable aspect of remifentanil is the predictability of its pharmacokinetics [23].

Remifentanil has a configuration very similar to the other piperidine derivatives, but it contains an ester linkage that makes it susceptible to metabolism by nonspecific esterases [23,25]. The esterase-based metabolism of remifentanil makes its pharmacokinetics largely independent of end-organ function, including renal or hepatic impairment [22,23]. This is in contrast to other opioids that exhibit considerable pharmacokinetic variability, as these depend on hepatic biotransformation and renal elimination. Due to esterase-based metabolism, there is a very rapid decline in drug concentration and the effect is likely to prevent any delayed respiratory depression after the administration of this drug during anesthesia, even when given at relative concentrations several times greater than those presently used with other opioids [28]. Similarly, its analgesic effect will terminate rapidly; thus, if analgesia is required, the clinician will have to plan to provide pain relief for the patient before discontinuing the drug [14,28]. The principal metabolite of remifentanil, remifentanil acid, is primarily excreted by the kidneys [22,23].

The pharmacokinetics of remifentanil, like many other drugs, have primarily been studied in adults and are less well defined in neonates. Importantly, they only display very limited maturation (ontogeny), and are already at an adult level of activity from birth onwards, so that metabolic elimination rather relates to weight, size or allometrics [39].

Four studies investigating the pharmacokinetics of remifentanil in children have been published. They demonstrate that pharmacokinetic data in children are comparable with those in adults [40,41,42]. However, until now no pharmacokinetic data have been available for remifentanil in preterm infants. Ethical restrictions on the volume of blood that can be collected for kinetic sampling largely preclude pharmacokinetic studies in preterm infants. Welzing et al. showed that very preterm infants exhibit a high nonspecific esterase activity in umbilical cord blood that is comparable with that of term infants. These results support clinical experiences that remifentanil is rapidly metabolized by preterm infants without prolonged side effects, as esterase activity is at an adult level of activity at birth [18,43].

The rapid onset of remifentanil is demonstrated by its brief half-time for equilibration between plasma and its effect compartment (CNS) (t1/2 ke0 of 1.0–1.5 min) [38]. This short t1/2 ke0, together with its rapid redistribution, results in a time to peak drug effect after a bolus of 1.5 min. In contrast, peak effects occur approximately 3–4 min with fentanyl and 20 min with morphine [40,42].

The rapid offset in concentration is best demonstrated by its context-sensitive half-time, which is extremely short 3–5 min regardless of the duration of infusion [23]. Thus, when the drug is administered within the appropriate clinical range, this very short context-sensitive half-time, combined with a rapid ke0, will result in a very rapid offset of opioid effect once administration of the drug has been terminated. The context-sensitive decrement line for remifentanil is time-independent [22].

Another distinctive pharmacokinetic characteristic of remifentanil is its rapid blood-brain equilibration time. The rapid clearance and blood-brain equilibration time enable remifentanil to act like a single-compartment drug with predictable plasma concentrations [28].

**Figure 1:**
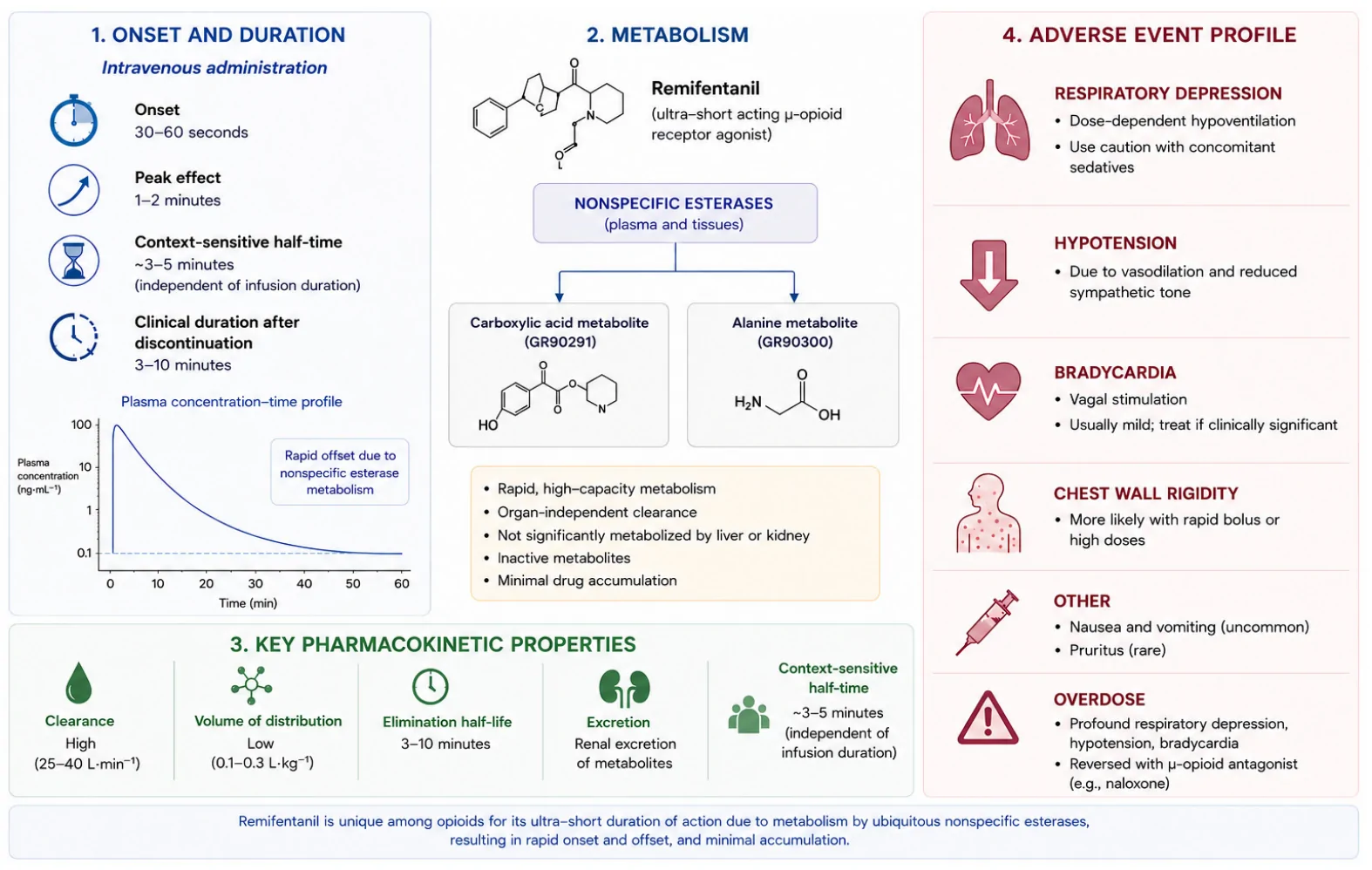
Remifentanil: pharmacokinetics and adverse event profile.

### 3.3. Pharmacodynamics of Remifentanil

The pharmacodynamics of remifentanil are closely related to its mechanism of action as a µ-opioid receptor agonist, and its effects are similar to those of other opioids, though with some distinct characteristics due to its rapid onset and offset of action [28]. Given the non-organ-dependent elimination with sole dependence on plasma and tissue esterases, the pharmacodynamic parameters are similar across all age ranges, including neonates [23,38].

#### 3.3.1. Effects

Remifentanil provides potent short-acting analgesia. In addition to its analgesic effects, remifentanil has sedative properties. It can cause mild to moderate sedation at lower doses and deep sedation or unconsciousness at higher doses, especially when used in combination with other anesthetics. The offset of remifentanil is quick and dramatic, with the loss of effective analgesia occurring within minutes of infusion cessation [23,28].

#### 3.3.2. Side Effects

Like other opioids, remifentanil can cause dose-dependent respiratory depression by acting on the brainstem, where it decreases the respiratory drive and responsiveness to carbon dioxide [28]. This is one of the most critical side effects, requiring careful monitoring during its use, especially in high doses or in sensitive populations (e.g., neonates, the elderly, or those with pre-existing respiratory conditions). If required, remifentanil’s respiratory depressant effect can be reversed by naloxone [28,39]. Opioids, including remifentanil, can cause bradycardia through their effects on the vagal nerve [44,45]. Remifentanil tends to cause less of this effect than other opioids (such as fentanyl) because of its more rapid onset and offset, but it still needs monitoring.

High doses or rapid (bolus) administration of remifentanil may induce muscle rigidity, particularly affecting the chest wall and diaphragm, which can compromise ventilation [23]. Although this phenomenon is more commonly associated with opioids such as fentanyl, caution remains warranted when using remifentanil. A study by De Kort et al. demonstrated that rapid infusion of remifentanil (less than 30 s) increases the risk of muscle rigidity; therefore, administration over a longer duration, such as approximately one minute, is recommended to reduce this risk [33].

Like all opioids, prolonged use of remifentanil can lead to tolerance and physical dependence. However, due to its short duration of action, these concerns are less common in typical clinical use.

There is increasing recognition that the long-term effects of opioid exposure, particularly in early life, are heterogeneous and remain incompletely understood. Evidence from preclinical and clinical studies suggests that prolonged or repeated exposure to opioids, especially at high doses, may be associated with alterations in pain sensitivity, stress responses, and neurocognitive development later in life. These effects are thought to arise from interactions between opioids, the developing central nervous system, and concurrent stress or pain exposure, making it difficult to disentangle causality. Long-term outcomes are influenced by multiple factors, including cumulative dose, duration of exposure, and the clinical context in which opioids are administered. Importantly, the available evidence primarily concerns prolonged or repeated administration, such as in neonatal intensive care settings [10,46].

Compared with longer-acting opioids, remifentanil has a faster onset and a faster time to reach the equilibrium between the plasma level and the brain concentrations [28].

There is a concern that opioids like remifentanil may impact neurodevelopment in neonates, particularly when used for prolonged periods of time. Remifentanil causes a dose-dependent suppression of the EEG [23]. Seizures have not been reported during the administration of remifentanil to humans [23].

For short-term, single-dose use of remifentanil—for example during procedures like endotracheal intubation—clinically relevant long-term adverse neurodevelopmental effects are considered unlikely, given its rapid metabolism [23,25], lack of accumulation [28], and minimal duration of central nervous system exposure [23]. Nonetheless, given the vulnerability of the developing brain, cautious use and further research into even brief opioid exposures remain warranted [10,46].

### 3.4. Studies Specifically Evaluating Remifentanil for Neonatal Intubation

Clinical studies of remifentanil in neonates are predominantly small, single-center investigations involving preterm and term infants undergoing elective intubation or INSURE. The strongest direct evidence comes from a small number of randomized trials, supplemented by pilot and observational studies.

Clinical characteristics and key outcomes of these studies are provided in Table 2 [11,14,15,30,31,32,33,34].

Pereira e Silva et al. (2007) reported better intubation conditions with remifentanil than with morphine in 20 preterm neonates [15]. Welzing et al. (2009) found feasible INSURE conditions with 71% first-attempt intubation and rapid extubation [31].

Smith et al. (2010) provided dose-finding data in infants and young children, with satisfactory conditions in most infants aged 0–3 months [32].

Choong et al. (2010) compared remifentanil with fentanyl plus succinylcholine in 30 hemodynamically stable neonates [30]. Physiological responses and median time to successful intubation were not significantly different between groups, while intubating conditions were rated more favorably with fentanyl/succinylcholine. Chest wall rigidity occurred in 2 of 15 infants receiving remifentanil. Thus, the study did not demonstrate clear superiority of remifentanil, but it also showed broadly comparable physiological responses [30].

Badiee et al. (2013) reported lower procedural pain scores with remifentanil during INSURE without significant differences in intubation duration, number of attempts, or oxygen desaturation; chest wall rigidity and laryngospasm were nevertheless observed [11]. Avino et al. [10] found remifentanil non-inferior to morphine-midazolam for intubation quality, with no significant difference in intubation attempts or overall adverse events [11]. Avino et al. (2014) found remifentanil non-inferior to morphine-midazolam for intubation quality, with no significant difference in intubation attempts or overall adverse events [14].

De Kort et al. (2017) illustrate the opposite pattern: with rapid administration and dose escalation, adequate sedation was achieved in only 14%, 43% required additional propofol, and chest wall rigidity occurred in 43%, leading to early study termination [33]. Audil et al. (2018) reported acceptable INSURE use but frequent transient desaturation and occasional need for naloxone. Together, these studies indicate that efficacy and safety are highly context-dependent [34].

Across studies, the most consistent signal is that remifentanil can provide effective analgesia and facilitate intubation in selected neonates, while rapid recovery may be useful for INSURE. However, evidence of superiority over alternative regimens is inconsistent. Differences in gestational age and illness severity, elective versus INSURE procedures, dose and infusion duration, use of atropine, propofol or midazolam, use or omission of neuromuscular blockade, operator experience, and differing definitions of adequate sedation or intubation success likely contribute to the conflicting findings [11,14,15,30,31,32,33,34].

However, the evidence also highlights important safety considerations, notably the risk of apnea and chest wall rigidity, which appear to be strongly influenced by dosing strategies and the speed of intravenous administration. While most studies reported minimal severe adverse effects, conflicting findings-especially in preterm infants—underscore the need for standardized administration protocols (e.g., intravenous bolus), careful patient selection and available mitigation strategies in the event of chest wall rigidity [11,14,15,30,31,32,33,34].

Safety findings likewise vary. Chest wall rigidity and respiratory adverse events were uncommon in some slower-administration studies but prominent in the rapid-bolus study by de Kort et al. [26]. This pattern supports caution about administration rate, but the available data are insufficient to define a universally safe dose or infusion rate [11,14,15,30,31,32,33,34].

Across the neonatal remifentanil literature, comfort, stress, and intubation quality were assessed using structured but heterogeneous methods (see Table 3). The study by Smith primarily focused on airway conditions, using composite intubation scores based on jaw relaxation, vocal cord position, patient movement, and ease of tube placement, which were then graded categorically (e.g., excellent, good, or poor) [32]. Trials in preterm infants, including those by Pereira e Silva et al. and Badiee et al., more often incorporated validated behavioral sedation tools such as the COMFORT or COMFORT-B scale, supplemented by physiologic markers like heart rate, blood pressure, and oxygen saturation to estimate procedural stress [11,15]. Investigations highlighting safety concerns, such as De Kort et al., emphasized respiratory and neuromuscular adverse effects rather than formal scoring, while studies centered on procedural success, including Audil et al., Welzing et al., and Avino et al., combined behavioral comfort assessment with categorical airway scores and detailed cardiorespiratory monitoring [14,31,34]. Overall, despite variability in specific instruments, most studies evaluated three domains in parallel—behavioral comfort (often COMFORT-based), structured intubation conditions, and physiologic safety—providing a multidimensional assessment of remifentanil’s suitability for neonatal airway management.

### 3.5. Practical Considerations for the Use of Remifentanil in Neonatal Intubation

Based on the limited available evidence, remifentanil may be considered for selected non-emergency neonatal intubation, particularly when rapid recovery is clinically desirable.

#### 3.5.1. Use in INSURE and LISA Procedures

Direct comparison of the role of remifentanil in INSURE and LISA highlights an important difference in the balance between its advantages and limitations. In INSURE, remifentanil’s rapid onset, short duration of action, and rapid recovery are aligned with the procedural goal of transient intubation for surfactant administration followed by prompt return to spontaneous respiration. The clinical studies summarized in this review support this potential role: remifentanil was associated with acceptable or favorable intubating conditions and successful completion of INSURE, and in the observational study by Audil et al. it was associated with a higher rate of successful extubation than the historical morphine-based approach [31,34]. However, the INSURE literature also demonstrates that the same rapid onset can narrow the therapeutic window and increase the risk of apnea, oxygen desaturation, or chest wall rigidity, particularly when remifentanil is administered rapidly [33,34]. Thus, in INSURE, the principal clinical objective is to achieve sufficient analgesia and sedation for intubation while preserving rapid recovery after surfactant administration.

The situation is different with LISA, where the procedural objective is to administer surfactant while maintaining spontaneous breathing and avoiding endotracheal mechanical ventilation. The manuscript therefore supports a more cautious interpretation of remifentanil in LISA: evidence for its use in this setting is less well established, and its respiratory depressant effects could conflict directly with the aim of preserving spontaneous respiration [12,16]. In contrast to INSURE, where rapid recovery is a major advantage, the principal concern during LISA is that opioid-induced respiratory depression, apnea, or chest wall rigidity may compromise spontaneous breathing and make the procedure more difficult. This distinction suggests that remifentanil currently has a clearer evidence-based rationale for selected INSURE procedures than for LISA. If considered for LISA, its use should be individualized, with careful dose selection, close respiratory monitoring, and immediate availability of positive-pressure ventilation and advanced airway support. The limited evidence also reinforces the need for prospective studies directly comparing analgesic and sedative strategies for LISA, rather than extrapolating findings from INSURE studies.

#### 3.5.2. Use in Hemodynamically or Respiratory Unstable Neonates

Particular caution is warranted in clinically unstable neonates. Although remifentanil is generally associated with limited cardiovascular depression compared with some alternative sedative agents, episodes of bradycardia, apnea, chest wall rigidity, and severe oxygen desaturation have been reported [2,10,31]. The risk–benefit balance may be less favorable in infants, and alternative regimens may be preferable when prolonged support or avoidance of opioid-related respiratory depression is a priority.

#### 3.5.3. Importance of Provider Experience

The successful use of remifentanil is highly dependent on provider and team experience. Because of its rapid onset and narrow therapeutic window, procedural delays after drug administration may result in either inadequate sedation or excessive respiratory depression [11,12,13,14,15,16]. Units adopting remifentanil-based premedication protocols should ensure that personnel are familiar with its pharmacology, dosing, preparation, administration, and management of potential adverse effects. The most experienced airway provider available should ideally perform the intubation, as minimizing the number and duration of attempts is particularly important when using a short-acting opioid [18].

#### 3.5.4. Monitoring and Procedural Preparedness

Continuous cardiorespiratory monitoring is mandatory whenever remifentanil is administered for neonatal intubation. At a minimum, monitoring should include continuous pulse oximetry, electrocardiography, heart rate assessment, and observation of respiratory effort. Non-invasive blood pressure monitoring should be considered when feasible [1,5,45]. Given the risk of apnea and chest wall rigidity, clinicians should ensure immediate availability of effective bag-mask ventilation, airway rescue equipment, suction devices, oxygen, and personnel skilled in neonatal resuscitation before administration [2,38]. Because adverse effects may occur rapidly, remifentanil should not be administered unless airway management can proceed without delay.

#### 3.5.5. Concomitant Use of Atropine

The use of atropine prior to neonatal intubation is supported by several guidelines and may reduce procedure-related vagal bradycardia [1,5,32]. Many published remifentanil studies incorporated atropine as part of the premedication regimen, making it difficult to distinguish the independent effects of remifentanil from those of vagolytic prophylaxis [2,14,31]. Although evidence specifically evaluating atropine-remifentanil combinations remains limited, atropine may be particularly valuable in preterm infants and in situations where repeated laryngoscopy attempts are anticipated. Consequently, administration of atropine before remifentanil should be strongly considered unless contraindicated, specifically in neonates with pre-existing tachycardia or tachyarrhythmia [1,5,32].

#### 3.5.6. Concomitant Use of Neuromuscular Blocking Agents

The role of neuromuscular blockade with remifentanil remains uncertain. Current neonatal intubation guidelines generally support the use of neuromuscular blocking agents as part of premedication for non-emergent intubation because they may improve intubating conditions and increase first-attempt success rates [1,5,33,45]. However, most studies evaluating remifentanil in neonates have not routinely combined it with neuromuscular blockade, and direct comparative data are scarce [2,10,14,31]. Potential advantages of adding a neuromuscular blocker include improved laryngoscopic conditions and mitigation of opioid-induced chest wall rigidity. Conversely, paralysis removes spontaneous respiratory effort and may increase the consequences of failed extubation, particularly in very preterm infants.

An additional consideration is the marked difference in duration of action between remifentanil and commonly used neuromuscular blocking agents. While the clinical effects of remifentanil typically resolve within 5–10 min after discontinuation, the duration of neuromuscular blockade with rocuronium may persist for approximately 30–60 min, and may be even longer in preterm infants. As a result, there is a significant risk that an infant may remain paralyzed after the sedative and analgesic effects of remifentanil have dissipated, potentially resulting in awareness without adequate sedation unless additional medication is administered. If neuromuscular blockade is deemed necessary, rocuronium at a dose of 0.6–1.0 mg/kg intravenously may be considered, providing rapid onset and an intermediate duration of action.

Until further evidence becomes available, the decision to combine remifentanil with a neuromuscular blocking agent should be individualized based on patient characteristics, operator preference, anticipated airway difficulty, and local practice patterns.

#### 3.5.7. Summary of Practical Recommendations

Current evidence supports considering remifentanil in selected non-emergent neonatal intubation when administered by experienced teams under appropriate monitoring conditions. Particular caution is warranted in clinically unstable infants, during LISA procedures where preservation of spontaneous breathing is desirable, and in settings where immediate airway rescue capabilities are unavailable. Atropine may be a useful adjunct to reduce procedure-related bradycardia, whereas the routine addition of neuromuscular blockade remains an area of ongoing uncertainty requiring further investigation [1,5,31,45].

## 4. Discussion

Endotracheal intubation in neonates remains a critical and technically demanding procedure that requires optimal conditions to maximize procedural success while minimizing pain, physiological instability, and adverse events [3]. Premedication is widely recommended as part of neonatal airway management because it reduces procedural stress and improves intubating conditions.

Although analgesics constitute a key component of premedication regimens, sedatives and neuromuscular blocking agents are frequently administered in combination to achieve adequate analgesia, sedation, and muscle relaxation [5].

**Figure 2:**
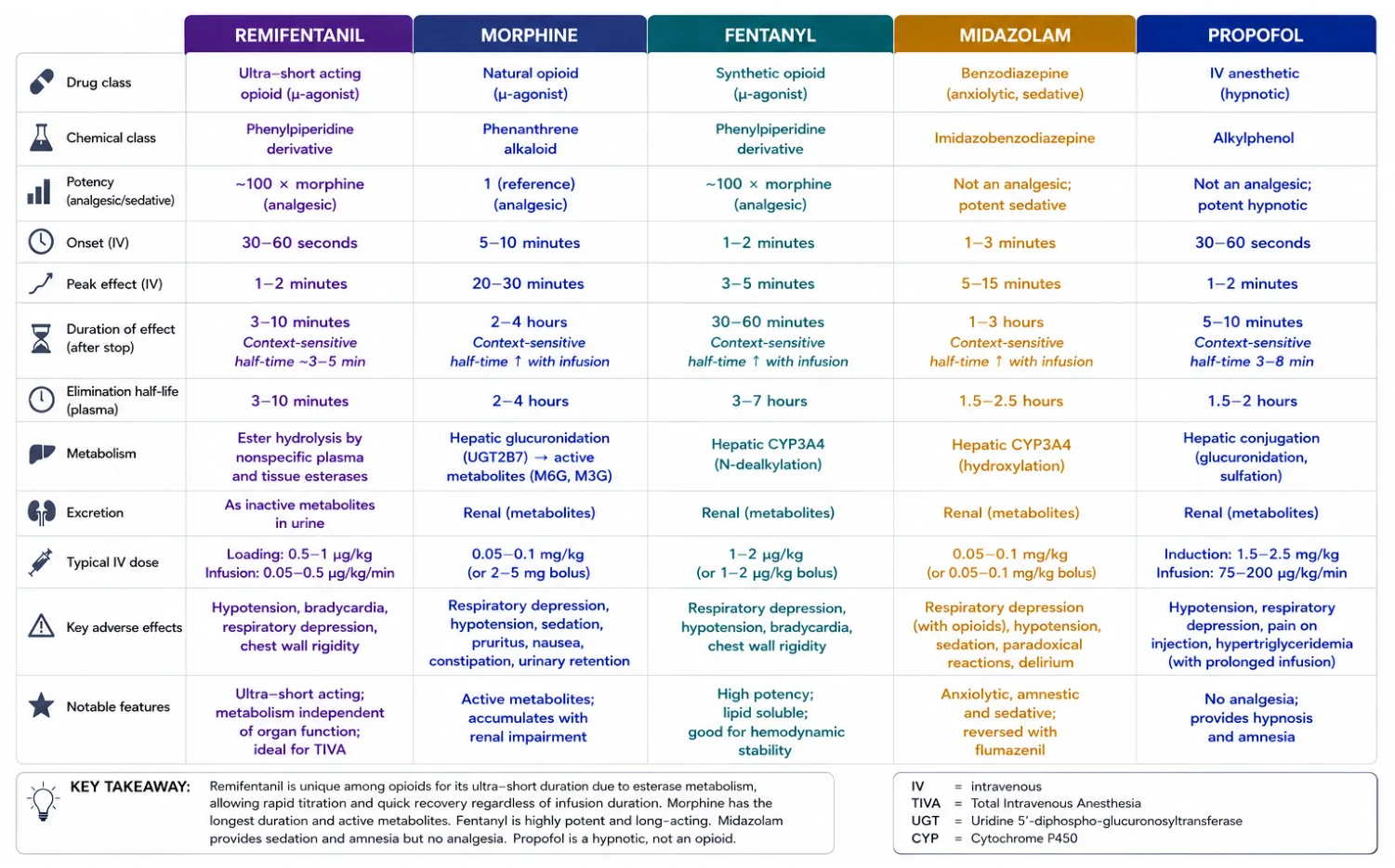
Comparative pharmacology: remifentanil vs. morphine, fentanyl, midazolam, propofol.

Given the wide range of pharmacological agents available for this purpose, a thorough understanding of their pharmacodynamic and pharmacokinetic characteristics is essential for selecting the most appropriate drug in different clinical scenarios. Factors such as onset and duration of action, metabolism, elimination, potency, and adverse effect profiles influence both efficacy and safety during anesthesia and procedural sedation. Figure 2 provides a comparative overview of remifentanil and several commonly used intravenous agents—morphine, fentanyl, midazolam, and propofol—highlighting their key pharmacological properties, clinical applications, and distinguishing characteristics. This comparison emphasizes the unique pharmacokinetic profile of remifentanil, particularly its ultra-short duration of action and organ-independent metabolism, while placing it in the broader context of commonly used anesthetic and analgesic agents.

Current evidence suggests potential usefulness in selected settings, especially when rapid recovery is desired, but it does not establish remifentanil as superior or routinely preferred.

The pharmacokinetic characteristics that contribute to its clinical utility may also present practical challenges. The brief duration of action may create a relatively narrow window during which optimal intubating conditions are achieved, potentially complicating airway management for less experienced providers [32]. Furthermore, the dose-dependent transition from analgesia to deeper sedation underscores the importance of careful titration and close monitoring during administration [24].

Remifentanil can be considered for selected non-emergent neonatal intubation and INSURE procedures when administered by experienced teams under appropriate monitoring conditions. Particular caution is warranted in clinically unstable infants, during LISA procedures where preservation of spontaneous breathing is desirable, and in settings where immediate airway rescue capabilities are unavailable [1,5,31,45]. The rapid onset of remifentanil, typically occurring within 60–90 s, may facilitate timely intubation and reduce the need for prolonged procedural attempts [47].

Uncertainty exists regarding the role of neuromuscular blockade when remifentanil is used for elective neonatal intubation. No adequately powered neonatal trials have definitively established whether remifentanil alone provides equivalent procedural conditions and safety outcomes compared with combined regimens.

Remifentanil should be used under close monitoring, particularly in neonates. The rapid onset of analgesia can be accompanied by an equally rapid onset of side effects if the dose given is too large or if it is given too rapidly [37]. Its narrow therapeutic window, potential for respiratory depression, and risk of chest rigidity underscore the importance of cautious administration and continuous monitoring. Respiratory depression is most often managed with naloxone, an opioid antagonist, which can reverse the effects of remifentanil if necessary [28,39].

Remifentanil causes a dose-dependent increase in the incidence and severity of muscle rigidity. Doses < 2 μg/kg given over 1 min have not been reported to cause rigidity. In a prospective study, de Kort et al. described chest wall rigidity in 6 patients (43%), and they discontinued the study prematurely due to side effects and a lack of efficacy [33]. Intravenous remifentanil was administered quickly and followed by a saline flush in approximately 30 s. Conversely, other studies have not reported on chest wall rigidity as a specific event with remifentanil. The absence of an objective scale to assess chest wall rigidity may partly explain the variability of its occurrence from one study to another, as its evaluation was subjective and operator-dependent.

Despite increasing clinical interest in the use of remifentanil in neonatal populations, the available evidence remains limited. While several studies have reported favorable intubation conditions with remifentanil, firm conclusions regarding its superiority over alternative premedication strategies cannot currently be drawn.

A major challenge in interpreting the literature is the overall quality and consistency of the available evidence. Many studies are observational, retrospective, or pilot investigations and are therefore susceptible to selection bias, confounding, and limited generalizability [11,40,41,42]. The overall quality of evidence should therefore be considered moderate to low. Although randomized controlled trials have been conducted [10,14,31], most studies remain relatively small, single-center investigations that are underpowered to detect uncommon adverse events or clinically meaningful differences in outcomes.

Furthermore, the pediatric remifentanil literature describes considerable variation in dosing and administration strategies, including differences in bolus dosing, continuous-infusion rates, timing of administration, and concomitant anesthetic agents [11,14,16,30,31,32,33,34]. Studies have used different doses, infusion rates, administration times, and combinations with adjunctive medications such as atropine and neuromuscular blocking agents, making direct comparison of outcomes difficult [11,16,39,40,41]. Assessment of procedural success has been hindered by the use of non-standardized sedation and intubation scoring systems, with varying definitions of adequate sedation, intubating conditions, and adverse events across studies [3,30].

Another important limitation is the scarcity of direct comparative studies. While remifentanil has been compared with morphine-based approaches in some investigations, robust head-to-head comparisons with other commonly used agents such as fentanyl, ketamine, propofol, or standardized opioid–neuromuscular blocker regimens remain limited [12,37,45]. As a result, current evidence is insufficient to establish the relative effectiveness or safety of remifentanil compared with alternative neonatal premedication strategies.

Most studies have focused primarily on short-term procedural outcomes, including intubation success, physiological stability, and immediate adverse effects. Long-term neurodevelopmental outcomes have rarely been assessed [8,34]. Given increasing awareness of the potential impact of early-life exposure to analgesic and sedative agents on neurodevelopment, this represents a significant gap in the literature. The absence of long-term follow-up data limits the ability to fully evaluate the safety profile of remifentanil in this vulnerable population.

## 5. Conclusions

This review provides a comprehensive evaluation of the available evidence on remifentanil for neonatal intubation, integrating pharmacological and clinical data, and provides a balanced discussion of both efficacy and safety outcomes. By synthesizing data from clinical studies, pharmacokinetic research, and contemporary guidelines, this review provides a clinically relevant overview of the potential role of remifentanil in neonatal airway management. In addition, it translates current evidence into practical recommendations for clinicians and highlights important gaps in knowledge that warrant further investigation.

In practice, remifentanil may be considered in experienced teams with continuous monitoring and immediate airway support, particularly for selected, specific, non-emergent intubation settings. Caution is warranted when spontaneous breathing must be preserved, as in LISA, and in unstable infants who may not tolerate opioid-related respiratory depression. Administration should be cautious rather than rapid, with readiness to provide positive-pressure ventilation and to manage chest wall rigidity or apnea. Other regimens may be preferable when established neuromuscular blockade, a different hemodynamic profile, or a more predictable procedural window is required.

The available literature remains limited by small sample sizes, methodological heterogeneity, inconsistent outcome reporting, and a lack of long-term safety data. Larger multicenter randomized controlled trials are still required to establish standardized dosing protocols, clarify the role of concomitant neuromuscular blockade, directly compare remifentanil with alternative regimens, and determine long-term neurodevelopmental safety outcomes.

Remifentanil should be administered by experienced clinicians in settings where continuous cardiorespiratory monitoring and immediate airway support are available. Slow and carefully titrated administration may reduce the risk of chest wall rigidity and severe respiratory depression. Clinicians should be prepared to provide positive-pressure ventilation and, when indicated, use neuromuscular blockade to facilitate intubation and manage opioid-induced rigidity. Standardized local protocols addressing dosing, administration technique, monitoring requirements, and management of adverse events are recommended to improve safety and consistency of care. Furthermore, careful documentation of efficacy and adverse events in routine clinical practice may help strengthen the evidence base while awaiting results from larger prospective studies.

The most important remaining questions concern optimal dosing and administration rate, the role of concomitant neuromuscular blockade, comparative effectiveness against commonly used regimens, and long-term safety. Larger multicenter trials using standardized outcomes are needed before stronger recommendations can be made.

We therefore encourage groups to report on their practices and outcomes, preferably based on prospectively collected data.

## Figures and Tables

**Table 1:** Summary of Quality Assessment. (**a**): Risk of bias assessment of randomized controlled trials (RoB 2) [35]. (**b**) Quality assessment of non-randomized studies (Newcastle–Ottawa scale) [36].

(**a**)				
**Study**	**Study Design**	**Assessment** **Tool**	**RoB 2 Domains** **Rated Low Risk**	**Overall RoB 2 Judgment**
Pereira e Silva et al. (2007) [15]	Randomized controlled trial	RoB 2	4/5	Some concerns
Choong et al. (2010) [30]	Randomized controlled trial	RoB 2	5/5	Low risk of bias
Badiee et al. (2013) [11]	Randomized controlled trial	RoB 2	5/5	Low risk of bias
Avino et al. (2014) [14]	Randomized non-inferiority trial	RoB 2	5/5	Low risk of bias
(**b**)				
**Study**	**Study Design**	**Assessment** **Tool**	**NOS Score**	**High Quality (≥7/9)**
Welzing et al. (2009) [31]	Pilot prospective study	NOS	6/9	No
Smith et al. (2010) [32]	Dose–response clinical study	NOS	8/9	Yes
de Kort et al. (2017) [33]	Prospective observational study	NOS	6/9	No
Audil et al. (2018) [34]	Prospective cohort study	NOS	6/9	No

**Table 2:** Studies on remifentanil in neonatal intubation. (**a**). Study characteristics. (**b**). Dosing regimens and administration strategies. (**c**). Intubation conditions and procedural outcomes. (**d**). Adverse effects and safety outcomes.

(**a**)			
**Study**	**Study Design**	**Number of** **Patients**	**Population**
Pereira e Silva et al. (2006) [15]	Randomized controlled double-blind trial	20	Neonates, 28–34 weeks’ gestation
Welzing et al. (2009) [31]	Prospective descriptive pilot study	21	Neonates, 29–32 weeks’ gestation
Smith et al. (2010) [32]	Dose-seeking study	20	Infants aged 0–4 months
Choong et al. (2010) [30]	Prospective double-blind randomized controlled trial	30	Preterm and term neonates
Badiee et al. (2013) [11]	Prospective case–control study	40	Neonates, 25–37 weeks’ gestation
Avino et al. (2014) [14]	Prospective non-inferiority randomized trial	Group 1: 36 Group 2: 35	Neonates ≥ 28 weeks’ gestation
De Kort et al. (2017) [33]	Prospective study	14	Neonates ≥ 27 weeks’ gestation
Audil et al. (2018) [34]	Retrospective review	62	Neonates, 31.6 ± 3.8 weeks’ gestation
(**b**)			
**Study**	**Premedication/Intervention**	**Administration Strategy**	
Pereira e Silva et al. (2006) [15]	Morphine 150 μg/kg + midazolam 200 μg/kg versus remifentanil 1 μg/kg + midazolam 200 μg/kg	Comparative premedication regimen	
Welzing et al. (2009) [31]	Atropine 10 μg/kg + remifentanil 2 μg/kg	Remifentanil administered over 1 min	
Smith et al. (2010) [32]	Glycopyrrolate 10 μg/kg + propofol 5 mg/kg + remifentanil 3.1–3.7 μg/kg	Dose-seeking regimen	
Choong et al. (2010) [30]	Atropine 20 μg/kg + remifentanil 3 μg/kg + saline placebo versus atropine 20 μg/kg + fentanyl 2 μg/kg + succinylcholine 2 mg/kg	Comparative randomized regimen	
Badiee et al. (2013) [11]	Atropine 10 μg/kg + 2 mL saline versus Atropine 10 μg/kg + remifentanil 2 μg/kg	Remifentanil administered over 2 min	
Avino et al. (2014) [14]	Group 1: atropine 20 μg/kg + midazolam 50 μg/kg + remifentanil 1 μg/kg; Group 2: atropine 20 μg/kg + midazolam 50 μg/kg + morphine 100 μg/kg	Comparative randomized regimen	
De Kort et al. (2017) [33]	Period 1: remifentanil 1 μg/kg bolus; Period 2: remifentanil 2 μg/kg bolus with escalation up to 5 μg/kg if needed	Fast bolus with saline flush over 30 s, repeat dosing permitted	
Audil et al. (2018) [34]	Atropine 10 μg/kg + remifentanil 2 μg/kg	Administration over 1–2 min, extendable up to 5 min	
(**c**)			
**Study**	**Intubation Conditions and Procedural Outcomes**		
Pereira e Silva et al. (2006) [15]	Significantly better intubation conditions in the remifentanil group (*p* = 0.0034). No significant differences in pain or stress levels before and after intubation.		
Welzing et al. (2009) [31]	Successful intubation on the first attempt in 71% and on the second attempt in 29% of cases. Intubation conditions were rated excellent in 67% and good in 33% of infants.		
Smith et al. (2010) [32]	Ideal intubation conditions achieved in 50% of infants and children receiving remifentanil doses of 3.1–3.7 μg/kg.		
Choong et al. (2010) [30]	Median time to successful intubation condition was not significantly different (247 vs. 156 s). Intubation conditions were rated more favorably with the fentanyl/succinylcholine regimen. First attempt intubation not reported.		
Badiee et al. (2013) [11]	No significant differences in time to endotracheal intubation (20.8 ± 6 vs. 22.8 ± 7.3 s) or number of attempts required for successful intubation.		
Avino et al. (2014) [14]	Poor intubation conditions at first attempt occurred in 25% of the remifentanil group versus 28.6% of the morphine-midazolam group (*p* = 0.471). Median intubation time at first attempt was 33 s (IQR 24–45) versus 36 s (IQR 25–59), respectively.		
De Kort et al. (2017) [33]	Adequate sedation achieved in only 2 patients (14%). Six patients (43%) required additional propofol to achieve adequate sedation.		
Audil et al. (2018) [34]	Overall safety considered acceptable during the procedure.		
(**d**)			
**Study**	**Adverse Effects and Safety Outcomes**		
Pereira e Silva et al. (2006) [15]	No significant differences in blood pressure or heart rate. No severe adverse effects observed.		
Welzing et al. (2009) [31]	Stable heart rate, blood pressure, and oxygen saturation. One case of hypotension reported. No chest wall rigidity observed.		
Smith et al. (2010) [32]	No adverse events reported.		
Choong et al. (2010) [30]	Chest wall rigidity in 2 remifentanil patients No significant differences in overall adverse events between groups		
Badiee et al. (2013) [11]	No significant differences in oxygen desaturation between groups.		
Avino et al. (2014) [14]	No significant between-group differences in hypotension, bradycardia, or adverse events.		
De Kort et al. (2017) [33]	High adverse event rate. Chest wall rigidity occurred in 6 patients (43%).		
Audil et al. (2018) [34]	Chest wall rigidity occurred in 4% of patients. One case of cardiopulmonary resuscitation following surfactant administration. Naloxone reversal was required in 5% of patients. Transient desaturation occurred in 34% of patients.		

**Table 3:** Assessment tools used in neonatal remifentanil intubation studies.

Author	Population	Comfort/Pain/Stress Scale	Intubation Condition Assessment	Additional Safety Monitoring
Pereira e Silva et al. (2006) [15]	Preterm and neonates	COMFORT score ± physiologic stress variables (HR, BP, SpO_2_)	Categorical intubation success score	Stress markers, hemodynamics
Welzing et al. (2009) [31]	Neonates	Behavioral comfort assessment ± physiologic parameters	Standard neonatal intubation scoring: jaw relaxation, cords, movement, ease of placement	Hemodynamics, oxygenation
Smith et al. (2010) [32]	Neonates and infants	Physiologic parameters ± behavioral observation (no standardized pain scale consistently reported)	Structured intubation score based on jaw relaxation, vocal cords, movement, ease of intubation	Apnea, desaturation, bradycardia
Choong et al. (2010) [30]	Preterm and neonates	Physiologic parameters (no validated pain, comfort or sedation scale used)	7-point Likert self-report scale	Hemodynamics, oxygenation return of spontaneous respiration
Badiee et al. (2013) [11]	Neonates	Behavioral comfort assessment ± physiologic parameters	No primary endpoint; procedure tolerance evaluated indirectly	Apnea duration, Ventilatory support
Avino et al. (2014) [14]	Neonates	Physiologic parameters ± behavioral observation (no standardized pain scale consistently reported)	Composite intubation score with categorical grading	Adverse events, apnea, recovery time
De Kort et al. (2017) [33]	Neonates	Sedation/behavior observation rather than validated scale	Not formally scored due to early termination	Chest wall rigidity, respiratory complications
Audil et al. (2018) [34]	Preterm and neonates (INSURE)	COMFORT or behavioral sedation assessment	Procedural success and extubation success rather than formal airway score	Desaturation, CPR events

## Data Availability

No new data were created or analyzed in this study. Data sharing is not applicable to this article.
